# A Random Field Excursion Model of Salt Induced Concrete Delamination

**DOI:** 10.6028/jres.099.045

**Published:** 1994

**Authors:** Gordon A. Fenton

**Affiliations:** Technical University of Nova Scotia, Halifax, Nova Scotia, B3J 2X4

**Keywords:** concrete degradation, de-lamination, level excursions, random fields, rehabilitation, reliability

## Abstract

Salt induced concrete delamination is a problem often encountered in parking garage slabs in northern climates where deicing salts are heavily used. A random field model of the delamination process is investigated and compared against some field data. Delaminated regions of the slab are modeled as excursions of a random field above a prescribed threshold and the growth of these regions with time is obtained by allowing the threshold to fall as a function of time. Simulation based excursion statistics are used to obtain the mean and variability of various aspects of the delamination process using this model.

## 1. Introduction

The delamination and spalling of concrete surfaces in parking structures in northern climates is an ongoing and expensive problem. The Transportation Research Board [1] estimates that between 50 and 150 parking structures in the Northeast and Midwest United States will need to be rehabilitated each year for the next 10 years at an average cost of $1 million per structure. Without proper design and/or maintenance, deicing salts brought in by vehicles from the roadways are deposited on the concrete surface along with water. Chloride ions gradually penetrate the concrete and electro-chemical processes lead to corrosion of the reinforcing steel. This results in both a degradation of structural integrity and, since the corrosion products occupy considerably more volume than the original steel, delamination or spalling of the concrete surface and loss of utility.

This paper is aimed at developing a tool to aid in a rational probabilistic approach to the rehabilitation of parking structures. Such an approach allows the optimal allocation of limited resources to this ongoing and rather expensive maintenance issue. A simple stochastic model involving only a few parameters is used herein to represent what is known to be a complex phenomenon. The following factors are suggested by Public Works Canada [2] to have the largest effect on the onset of concrete delamination;

- *chloride ion input:* quantities of deicing salts used,
- *concrete permeability:* influenced in turn by water/cement ratio, intensity and frequency of cracking, surface coatings/sealers and construction practices,
- *ambient temperature, humidity, precipitation*
- *concrete cover depth*
- *pH of local aggregates*
- *conductivity:* wetness of concrete.

It is immediately recognized that virtually all of these factors are highly variable from structure to structure and within a single structure from point to point. As well, on a practical basis for an existing structure, some of the factors are unknowable except through extensive destructive testing. Clearly a representative model should not require experimental validation on a per structure basis, where the experimentation may be more expensive than the final repairs. This, in fact, is the primary motivation for the use of stochastic models.

In a different approach to the same problem, Attwood et al. [3] develop a limit state function for parking structures based on a critical fraction of floor area delamination, *D_α_*,

$$m=D_{\alpha }-\alpha S\left(t-t_{1}\right),t>t_{1},$$ (1)

where *m* ≤ 0 denotes the “failure” state, *S* is the annual delamination rate, *α* is a correction factor accounting for ambient temperature and crack widths, *t* is time in years, and *t*_1_ is the time to initiation of delamination (also in years). Note that the second term is taken to be zero for all *t ≤ t*_1_. Attwood et al. employ a First-Order Reliability Method (FORM) to estimate reliabilities associated with the delamination of a parking garage structure. In their approach, all of the factors appearing in Eq. (1) are expressed in terms of random variables having assumed distributions and the joint cumulative probabilities are evaluated using FORM.

In this paper a 2-dimensional random field model representing the spatial delamination process over time is investigated. The random field may be loosely interpreted as the out-of-plane stress field at the reinforcement level (which changes randomly from point to point over the area of the slab). Delaminated regions are represented by excursions of the random field above some threshold which can be thought of as a critical concrete resistance to horizontal splitting. In fact the stress field itself will not be considered in that it is unmeasurable in practice. Rather, the field excursions will be used to represent the delaminated regions directly, since these regions are measurable to some extent. A simple random field model is adopted whose primary motivation is to attempt to shed light on the following questions;

1. what is the mean and variability of the total area of delamination as a function of time?
2. what is the average size of individual delaminated regions as a function of time?
3. how many delaminated regions can be expected in a slab?

These are essentially questions regarding the statistics of excursion regions and so some simulation results regarding excursion statistics will be presented in the next section.

**Fig. 1:**
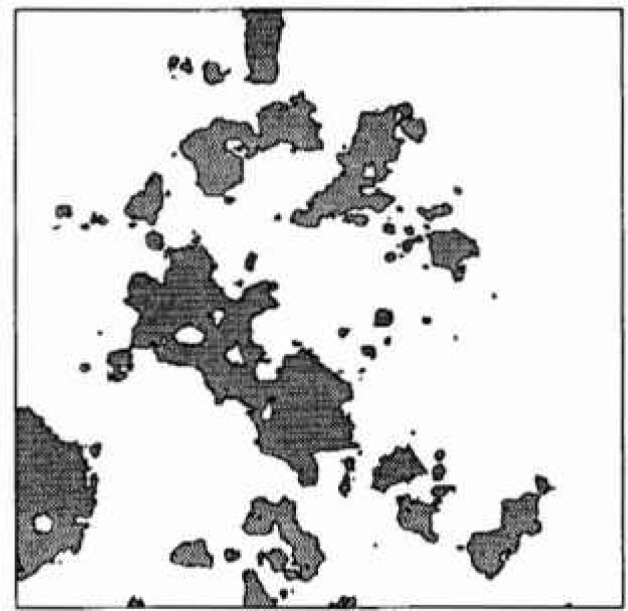
Excursions of a two-dimensional random field above threshold *bσ.*

Figure 1 illustrates an example of excursions of a random field above some predefined threshold. In the context of this paper, the dark regions can be viewed as areas where concrete delamination has occurred at some fixed time. As time progresses, delaminated regions are expected to grow in size, corresponding to a falling threshold level. A falling threshold is equivalent to a rising mean, in the case of a homogeneous field. Since the excursion statistics in the next section are developed as functions of the threshold, the falling threshold interpretation is used here rather than a rising mean. In either case, a nonhomogeneous field could be employed, if the data so indicated, by considerably extending the simulation based study (in the absence of analytical results). In this preliminary investigation, only a homogeneous field is considered.

To represent the delamination process, excursions of an isotropic Gaussian random field having mean zero and unit variance will be used. Although other values of mean and variance are possible, the excursion statistics are dependent purely on the distance between the mean and the threshold. This distance can be expressed in units of *σ*, the standard deviation, so that there is no advantage in choosing anything other than a mean zero, unit variance field. The quantity *bσ* will be referred to henceforth as the physical threshold and *b* alone as the threshold. The choice of an isotropic Gaussian process has been made largely for simplicity, there being little evidence available to clearly justify other types of random functions.

## 2. Excursion Statistics in Two Dimensions

With respect to excursion statistics, such as the mean number and area of isolated excursions, analytical results developed to date are asymptotic in nature, accurate only at very high thresholds where the excursion process approaches a Poisson point process. Often in engineering problems the interest is in thresholds which are quite a bit lower, such as the delamination process considered herein. In order for the proposed random field model to be useful in this context, excursion statistics should be available. This section summarizes a study in which excursion statistics are obtained through Monte Carlo simulation. Specifically, an ensemble of 2000 realizations of an isotropic zero mean, unit variance Gaussian random field, *Z*(*x*), with Markovian covariance function,

$$B\left(\tau \right)=\sigma^{2}\rho \left(\tau \right)=\sigma^{2}\exp\left\{-\frac{2}{0}\left|\tau \right|\right\},$$ (2)

are produced using the Local Average Subdivision (LAS) method [4, 5], where *τ* is the lag vector and *θ* is the scale of fluctuation. The scale of fluctuation is loosely interpreted as the distance over which correlation is significant. Since many of the statistics of interest depend strongly on the scale of fluctuation, 2000 realizations were generated at each of 5 different scales of fluctuation.

Individual realizations are decomposed into excursion regions and “holes,” using a space-filling algorithm, over a range of thresholds *b* =[−4, 4]. The mean and variance statistics of the excursions are estimated over the ensemble. It should again be emphasized that *b* is measured in units of standard deviation, for example *b* = 2 implies a threshold at two standard deviations from the mean. For a unit variance field there is no distinction between the value of *b* and the physical threshold level. However the local averaging performed by the LAS method results in a slight decrease of the variance of the discretized field, as dictated by local averaging theory. In that each field is represented as a discrete lattice of 128 × 128 “cells,” the variance of each cell varies from 0.971 at the smallest scale of fluctuation considered to 0.999 at the largest. The distinction between cell variance and point variance will be ignored in this paper, although the results presented in the various plots to follow are accurate in this respect. For a more rigorous treatment of this issue, see Ref. [6].

Within a given domain *V* = [0, *L*_1_] × [0, *L*_2_] of area *A_t_ = L*_1_ *L*_2_, the total excursion area per unit area, *D_b_*, where the process *Z*(*x*) exceeds some threshold, can be defined by

$$D_{b}=\frac{1}{A_{T}}\int_{V}I\left(Z\left(x\right)-b\sigma \right)dx,$$ (3)

where *bσ* is the physical threshold of interest, *σ*^2^ being the variance of the process, and *I*(•) is the indicator function defined on *V* (taken to be zero outside the domain *V*)

$$I\left(t\right)=\{\begin{array}{cc} 1 & \mathrm{if}\;t\geq 0\\ 0 & \mathrm{if}\;t<0 \end{array}.$$ (4)

**Fig. 2:**
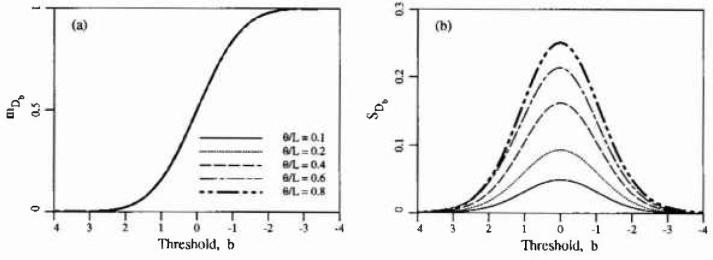
Estimated statistics of total excursion area per unit arca, *D_b_*,: a) mean, b) standard deviation.

For a homogeneous process, the expected value of *D_b_* is simply

$$E\left[D_{b}\right]=P\left[Z\geq b\sigma \right],$$ (5)

which, for a zero-mean Gaussian process yields

$$E\left[D_{b}\right]=1-\Phi \left(b\right),$$ (6)

where *Φ* is the standard normal distribution function. The estimate of *E*[*D_b_*], denoted $m_{D_{b}}$, derived using the simulation results is shown in Fig. 2 and is in complete agreement with Eq. (6). Figure 2 (b) shows the estimated standard deviation of *D_b_* denoted $s_{D_{h}}$. Note that while *E*[*D_b_*] is independent of *θ*, its variance is not. In keeping with the practice of normalizing all results, the scale of fluctuation has been normalized with respect to $L=\sqrt{A_{T}}$ in these plots. Note also that the horizontal threshold axis values decrease to the right—this is because the threshold axis is associated with time in the next section and time increases to the right as usual.

**Fig. 3:**
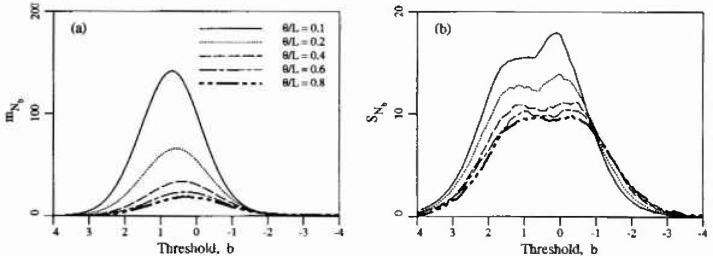
Estimated statistics of number of isolated excursions, *N_b_*: a) mean, b) standard deviation.

Figure 3 shows the estimated mean and standard deviation of the number of isolated excursion regions, *N_b_*, denoted $m_{N_{b}}$ and $s_{N_{b}}$ respectively, as a function of scale and threshold, Although not readily apparent from the plot, the limiting value of $m_{N_{b}}$ at the right edge of the plot (*b* → − ∞) is 1. In other words, a single excursion exists over the entire domain for very low thresholds. At the other extreme (*b → ∞*), the limiting value is zero as expected. Although the roughness of the estimate $s_{N_{b}}$, as seen in Fig. 3(b), is as yet unexplained, it appears unlikely that it arises from statistical uncertainty in the estimation procedure. Based on a sample size of 2000 realizations, a 90 percent confidence bound on the standard deviation of *N_b_* is about $\pm 0.025s_{N_{b}}$ which is smaller than the size of most of the “bumps” seen in Fig. 3(b).

Within a given realization, the average area of an isolated excursion per unit domain area, *D*_c_, can be obtained using the number of excursions,

$$D_{e}=\frac{D_{b}}{N_{b}}.$$

Since *D_b_* is the sum of the *N_b_* isolated excursion areas, the expected value of *D*_e_ is just

$$E\left[D_{e}\right]=\frac{E\left[D_{b}\right]}{E\left[N_{b}\right]}$$

**Fig. 4:**
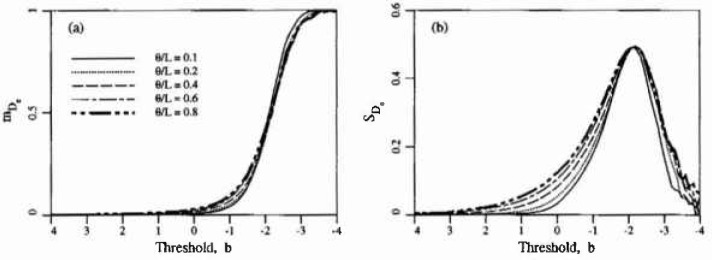
Estimated statistics of isolated excursion areas per unit area, *D_c_*,: a) mean, b) standard deviation.

Figure 4(a) illustrates this result using the estimated mean value of *N_b_* shown in Fig. 3(a). The estimated standard deviation of *D*_e_ shown in Fig. 4(b) is derived using the assumption that the sizes of isolated excursions are independent. While this is true from realization to realization, it is clearly not true within a single realization. Thus Fig. 4(b) can only be considered to be a rough indication of the true variability of the area of isolated excursions.

## 3. Calibrating the Model

**Fig. 5:**
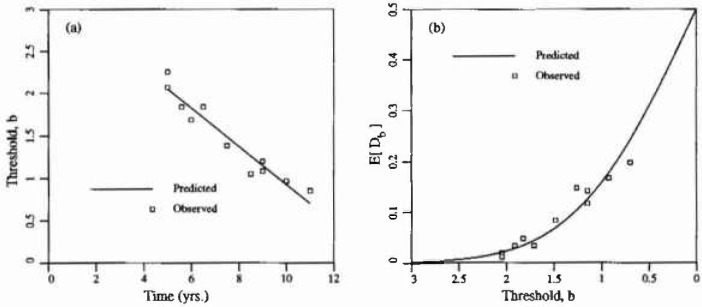
Regression results using Trow’s delamination rate data: a) regression, b) observed versus predicted *D_b_.*

There are essentially only two parameters in the random field model considered in this study. These are the scale of fluctuation, *θ*, and the threshold level, *b.* Although there is little published experimental evidence to allow a clear statement of what these parameters should be for a given structure, some preliminary estimates are possible. First the relationship between the threshold level and the age of the structure can be obtained by fitting delamination versus age data collected by Trow [7] and presented in Ref. [3] to the curve of Fig. 2(a). A good fit was obtained using least squares regression (*r*^2^ = 0.93) on the linear relationship *b* = 3.17 − 0.225*t*. To obtain this relationship, the total fraction of delaminated area observed by Trow, *D_b_*, was plotted against *b = Φ*^−1^(1 − *D_b_*) and a best fit line obtained. Figure 5(a) shows the regression results and Fig. 5(b) shows where the observed delamination results would appear on Fig. 2. To expand the scale, only positive thresholds are shown in Fig. 5(b). The choice of function relating time and threshold is largely arbitrary as long as *b*(*t*) is a decreasing function over all times of interest (assuming delaminated areas cannot “heal”). While a quadratic gives a slightly better fit to the raw data, it violates this principle and so cannot be used to extrapolate.

**Fig. 6:**
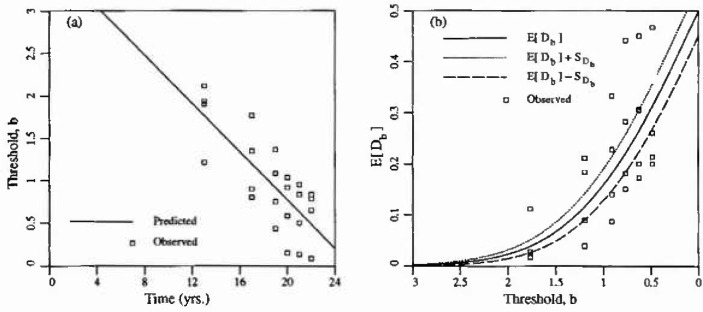
Delamination rate data provided by Public Works Canada. The fitted line is *b* =3.61 − 0.142*t* with *r*^2^ = 0.6.

Some additional unpublished data was made available to the author by Public Works Canada based on a survey of a single parking garage structure in Ontario, Canada. Figure 6 illustrates this data. The ±1 standard deviation curves are obtained using Fig. 2(b) with *θ/L* = 0.1. Clearly, for such a choice in *0*, the variability in *D_b_* predicted by the random field excursion model underestimates the variability in the observations. The additional variance arises because the time-threshold relationship *b*(*t*) is itself a function of random coefficients, as implied by the regression analysis. Alternatively and equivalently, the additional variability could be ascribed to the fact that the delamination field is not homogeneous on such a scale. When considered at the scale of a typical bay, some bays show much higher delamination rates than others, perhaps corresponding to smaller mean cover depths and/or higher Cl^−^ inputs. Note also that larger values of *θ*/*L* lead to higher standard deviations in *D_b_* so that the variability seen in Fig. 6 could also be explained by larger values of *θ.* However the choice of *θ* should not be based on the variability in *D_b_*, rather this observation indicates that perhaps the Gauss-Markov covariance model is not appropriate.

**Fig. 7:**
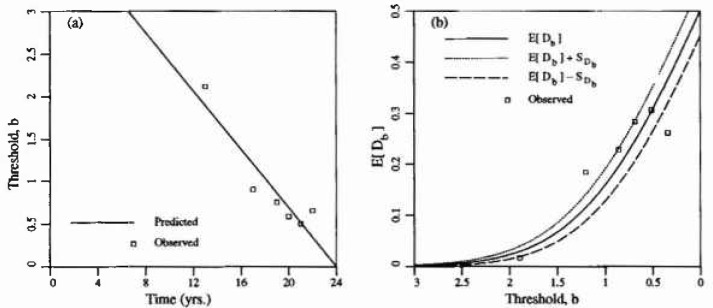
Delimination rate provided by Public Works Canada over a single slab region.

In this study, *b*(*t*) will be treated as a deterministic function which corresponds to the choice of a homogeneous random field. It is beyond the scope of this initial investigation to consider estimating the parameters of a nonhomogeneous (or self-simitar) field to represent the delamination process generally, although this appears indicated. Since plan views of the delaminations are available from Public Works for individual bays of approximately 5m × 5 m in extent (*L* =5), attention will be restricted to data on such a scale over which the field can be considered homogeneous. Choosing one such area from the Public Works data, the measured delamination fraction as a function of time is shown in Fig. 7. In this case the line of best fit was found to be

b=4.13−0.172t (7)

with *r*^2^ = 0.86. Notice that for such a case, the variability in the observations is substantially reduced and that 2/3 of the observations lie on or within the ±1 standard deviation curves for *0/L* = 0.1 (see also Fig. 5). This result appears encouraging although it is recognized that it could be due in part to the reduced number of samples, even though similar results were found for most other bays.

Before considering the estimation of *θ*, it is worth pointing out a further difference between the random excursion model and the commonly accepted delamination model. Salt induced reinforcement corrosion is generally believed to involve two stages (see Ref. [8]): 1) an initiation phase during which the alkalinity of the concrete surrounding the reinforcement (which renders the steel passive) is reduced by the migrating Cl^−^ ions, and 2) an active phase during which corrosion takes place. Attwood et al. [3] estimates the initial phase to last 4.7 years. During this phase no corrosion is assumed to take place. Note that such a model can only be applied to points in the slab where the concrete is in contact with the reinforcement.

In contrast with this two stage model, the random field excursion model admits some probability of delamination even at time *t* = 0. Using Eq. (7), one obtains *b* =4.13 at time *t* = 0, so that the expected total delamination area per unit area is 1.7 × 10^−5^ or about 17 mm^2^/m^2^ of slab. At these levels the precise definition of delamination comes into question. If it is strictly interpreted as a loss of bond between the concrete and reinforcement then this result is not unreasonable given the presence of cracks, voids and the initial state of the reinforcement. At time *t* = 5 years, the expected total delamination area is still only about 6 cm^2^/m^2^ of slab, a level which is probably still largely undetectable at the surface of the slab and presumably would correspond to corrosion in the immediate neighborhood of surface cracks. Although it is believed that cracks do not contribute significantly to the areal delamination process [8], it is not unreasonable to expect that they can be initiators of the corrosion process at discrete points in the slab. If this is the case, then the many year delay before the onset of observable levels of delamination implies that the corrosion growth should be quite slow at first. The results predicted by Fig. 2(a) are in basic agreement with this in that *E*[*D_b_*] grows very slowly for *b* decreasing to about 2 [corresponding to *t* < 12 years using Eq. (7)].

**Fig. 8:**
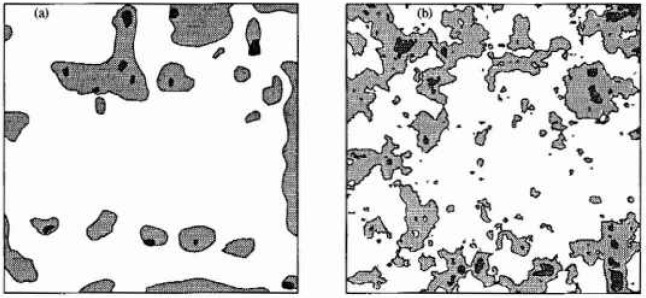
a) Observed delamination regions at ages *t* = 13 years (dark grey) and *t* = 22 years (light grey) on a 5m × 5m portion of a parting garage slab. b) Excursions of a random field above *b* = 0.34(*t* = 13) and *b* = 19(*t* = 22) using *θ* = 0.5 and Eq. (7).

**Fig. 9:**
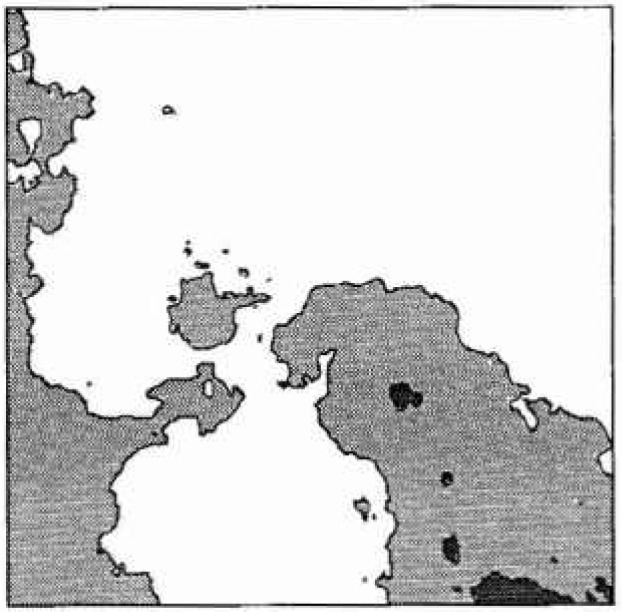
A realization of excursions at *t* = 13 years (dark grey) and at *t* =22 years (light grey) using *θ* = 4 m on a 5 m × 5 m field.

Turning now to the estimation of the scale of fluctuation, *θ*, it becomes apparent that this task is complicated by the type and quality of data available. Ideally, one would take measurements of the corrosion induced stress field over a number of structures, estimate a spatial covariance structure and from this obtain *θ.* Even if this approach were possible, the nonhomogenieties mentioned above would make it difficult. However, in general the stress field is unmeasurable and what little data is available generally consists of surveys giving the spatial extent of delaminated regions. Figure 8(a) illustrates such a survey while Fig. 8(b) is a realization of the random field excursions using *θ* = 0.5 m. Once the time-threshold relationship has been established, and for the purposes of this argument Eq. (7) will be used, a possible technique of estimating *θ* would be to count the average number of excursions and enter Fig. 3(a) at the appropriate threshold to estimate *θ*/*L*. Purely on the basis of Fig. 8(a) this yields estimates of *θ* = 2 to 4 m (*θ*/*L* = 0.4 to 0.8). However realizations at this scale yields excursions which are generally far too well connected as shown in Fig. 9. Realizations at such large scales appear like large land masses with many small islands close to shore. Figure 8(a) has “islands” that are more uniform in size and distribution implying a smaller scale of fluctuation. In Fig. 8(b), produced using *θ* = 0.5, the larger islands are of similar size to the delaminations seen in Fig. 8(a). This along with arguments to follow supports the choice of a smaller scale of fluctuation.

While one cannot expect the plots in Fig. 8 to be identical since they are both independent realizations of a random process, any more than one could expect the pattern of delaminations in another building to be identical, a number of points can be made about the two plots;

1. there is no apparent spatial orientation of the delamination regions in the observations of Fig. 8(a), indicating that the assumption of isotropy is acceptable, at least for this case.
2. about half of the excursions in the random field model (Fig. 8b) are of very small extent. This fraction increases at larger scales. On the other hand, in Fig. 8(a) there are only very few “small areas appearing in the later survey. It seems reasonable to suspect that additional small delamination regions are in fact occurring in the real slab but that the chain-drag surveying technique is unable to resolve them. Operator bias will almost certainly also be present due to the prior knowledge of existing delamination areas.
3. the random field model is much “rougher” than the observed delamination plots. Again this is likely a problem with the ability of the chain-drag survey to resolve detail.

The last two points illustrates the difficulty in estimating *θ* on the basis of delamination surveys. The chain-drag method depends on setting up reverberations in the delaminated concrete. For delamination details below a certain size, the frequency shifts are undetectable to even the most sensitive human ears, rendering them unnoticeable. Thus many of the delamination details on which an estimation of *θ* depend are unavailable using current surveying techniques. To some extent the deficiencies in the chain-drag procedure could be accommodated simply by introducing more local averaging in the random field model — effectively smoothing the field. While such a “correction” would not likely result in an improved delamination model, it may allow improved estimates of *θ*. For the purposes of this investigation, the realization based estimate of *θ* = 0.5 m is used.

## 4. Discussion

Conceptually the model proposed herein is quite attractive in that the delamination process is indeed a threshold excursion process in two dimensions. Once the details of the model have been established (type of distribution, time-threshold relationship and scale of fluctuation) and some of the properties of threshold excursions in two dimensions have been determined analytically or via simulation, the model can be used in a reliability context. For example, using Eq. (7), at time *t* = 12 years (*b* = 2.07) Fig. 2 along with Eq. (6) indicates that $m_{D_{b}}=0.0194$ and $s_{D_{b}}=0.0080$, using *θ* = 0.5 m. If *D_b_* is assumed to have a Beta distribution, which is properly bounded between 0 and 1, then the probability that *D_b_* is less than 3 % at 4 years is given by

$$P\left[D_{b}\leq 0.03\right]=0.90.$$ (8)

In turn, if a target reliability of 0.9 were chosen against delamination in excess of 3 %, then inspection of the garage would be recommended at *t* = 12 years for this structure.

Because the model includes the spatial aspects of delamination, it can be used to evaluate testing and mapping procedures. For example, at time *t* = 12 years one could expect about 42 isolated excursions on a 5 m × 5 m slab (*θ* =0.5 m) with a standard deviation of about 11. The area of each isolated excursion would average about 0.011 m^2^ with a standard deviation of about 0.022 m^2^. Surveys which yield results considerably different than these may require verification using other techniques. In addition, the spatial description of delamination could be used in a structural reliability study. For this the simulation approach could be employed to yield a measure of the degree of clustering of the delamination regions (see Ref. [6]).

One recognizes that the excursion model is attempting to predict the cracked state of a concrete slab. In that internal cracks are exceedingly difficult to map, even in controlled laboratory conditions, the model is to some extent intuitive and will likely remain so until improved surveying techniques are developed. Nevertheless the model demonstrates some promising features and can be used as a powerful reliability tool when its parameters are clearly defined in terms of additional data and simulation studies. In particular, the data shown in Fig. 6 indicates that perhaps alternative correlation functions [see Eq. (2)] should be studied — multiple-scale or self-similar type random fields are suggested, showing the small scale behaviour over small regions while reflecting also the large scale, slower variations over larger domains. Also the fact that the model allows delamination to occur at time *t* = 0 (or before) implies that some thought should be given to the assumption of a Gaussian random field and/or the time-threshold relationship. In the interim, however, the choice of a Gaussian field and linear time-threshold relationship leads to results which appear reasonable for any time *t* > 0.

## References

[b1-jresv99n4p475_a1b] 1Transponation Research Board, Highway Deicing, Special Report 235, Washington, DC (1991).

[b2-jresv99n4p475_a1b] 2Public Works Canada, Developmont of Reliability-Based Methods for Cost Effective Maintenanee of Parking Structures, Technical Report, Ottawa (1989).

[3] Attwood D, Nessim MA, Ghoneim A, Cormeau A, Cheung MS (1991). Applicalion of reliability theory to in-service monitoring and maintenance of parking garages. Can J Civ Eng.

[4] Fenton GA (1990). Simulation and Analysis of Random Fields. PhD Thesis.

[5] Fcnton GA, Vanmarcke EH (1990). Simulation of Random Fields via Local Average Subdivision. ASCE J Eng Mcch.

[6] Fcnton GA, Vanmarcke EH (1992). Simulation-based excursion statistics. ASCE J Eng Mech.

[7] Trow Ltd. Consulting Engineers (1984). Parking Structure Deterioration: A Survey and Analysis of its Extent and Influencing Factors.

[8] Becby AW (1983). Cracking, cover, and corrosion of reinforcement. Concrete International.

